# Garidisan: Improving the Quality of Ulcer Healing in Rats with Ulcerative Colitis

**DOI:** 10.1155/2017/8721257

**Published:** 2017-08-08

**Authors:** Lin Wang, Wei-Zhi Liu, Ling-Yan Pei, Yu-Shi Ke, Jian Cui, Shu-Chun Li

**Affiliations:** ^1^Institute of Chinese Minority Traditional Medicine, Minzu University of China, Beijing 100081, China; ^2^Department of Histology and Embryology, Xinxiang Medical University, Xinxiang, Henan 453003, China; ^3^Key Laboratory of Chinese Minority Traditional Medicine, Minzu University of China, State Ethnic Affairs Commission and Ministry of Education, Beijing 100081, China

## Abstract

Garidisan, commonly used in Mongolia to treat ulcerative colitis (UC), contains wild poppy and* Artemisia frigida *Willd. Clinical evidence shows that Garidisan can effectively treat UC and that recurrence is low. Thus, we evaluated the effects of Garidisan on ulcer healing quality and the regulation of immune balance in rats with experimental UC. UC was induced by immunization with TNBS and Garidisan significantly reduced DAI, CMDI, and HS. H&E staining, SEM, and VG staining showed that Garidisan repaired damaged intestinal mucosa and significantly reduced expression of ICAM-1 and CD105 in regenerated tissues of UC rats. Collagen fibers were significantly fewer as well after treatment. Garidisan elevated EGF, VEGF, bFGF, VEGFR2, and FGFR1 of UC rats, reduced CD3^+^CD4^+^/CD3^+^CD8^+^ T cell ratios, and increased CD4^+^Th1/CD4^+^Th2 cell ratios and IFN-r/IL-4 ratios in peripheral blood of UC rats. In conclusion, Garidisan promoted tissue maturation of regenerated tissues by regulating the immune balance and improved functional maturity of regenerated tissues by reducing collagen formation, promoting maturation of new blood vessels, and increasing expression of growth factors and their receptors.

## 1. Introduction

Ulcerative colitis (UC) is a chronic, nonspecific inflammatory disease involving mainly the rectum and colonic mucosa, but it may involve the entire colon mucosa. Clinical manifestations of UC include diarrhea, abdominal pain, and purulent stools. UC often recurs after healing [1]. The pathogenesis of UC has not been clearly identified and may be associated with the environment, intestinal symbiotic bacteria disorders, damage of the epithelial barrier, genetic susceptibility, and immune disorders [2–4]. Studies indicate that regulatory and helper T cell imbalances [5–7] as well as anti-inflammatory and proinflammatory cytokine imbalances occur in UC patients [8, 9], suggesting that immune disorders contribute to the pathogenesis of UC.

Immunosuppressive agents, anti-inflammatory drugs (e.g., 5-amino salicylic acid, corticosteroids, and antibiotics), and biological agents (e.g., TNF-*α* antibodies) have been used to treat UC and although combination drug therapies offer some efficacy, hypertension and fever are side effects, and the disease recurs easily after drug withdrawal [1, 10, 11]. Thus, the development of specific treatments with fewer side effects that prevent UC recurrence is of interest.

Plant and plant-based drugs have been used to treat various diseases since recorded history, but few current studies support any certain efficacy. Garidisan, often used to treat UC in Mongolia, China, contains wild poppy (*Papaver nudicaule *L.) and* Artemisia frigida *Willd. Clinical evidence allegedly suggests that Garidisan can treat UC with fewer side effects, and UC patients treated with Garidisan are reported to have fewer recurrences [12, 13], but how this occurs is unclear. Studies indicate a relationship between UC recurrence and the quality of ulcer healing. In 1993, Tarnawski's group proposed the concept of ulcer healing quality and considered that ulcer healing required mucosal and submucosal tissue repair and regeneration. Assessment of ulcer healing quality focuses on maturation of local mucosal structure regeneration and ulcer functions [14–16]. Hence, we used these criteria to evaluate Garidisan treatment of UC and to explore its therapeutic mechanism.

## 2. Materials and Methods

### 2.1. Animals and Grouping

Ten specific-pathogen-free (SPF) grade New Zealand rabbits (male : female = 1 : 1) and 90 SPF grade Wister rats (200 ± 10 g) each (male : female = 1 : 1) were purchased from and raised at Beijing Vital River Laboratory Animal Technology Co., Ltd. (Beijing, China, SCXK 2012-0001). All rats were randomized into seven groups: normal control (*n* = 12), UC model (*n* = 13), low-dose Garidisan (*n* = 13), moderate-dose Garidisan (*n* = 13), high-dose Garidisan (*n* = 13), sulfasalazine (SASP, *n* = 13), and Bupiyichangwan (*n* = 13) groups.

### 2.2. Mongolian Garidisan Composition and Preparation

Mongolian Garidisan, containing 15 g* P. nudicaule* L. and 24 g* A. frigid* Willd., were purchased from an herbal medicine procurement and supply company in Inner Mongolia and identified by Associate Professor Almaz, Institute of Chinese Minority Traditional Medicine, Minzu University of China, Beijing, China.* P. nudicaule* L. and* A. frigid* Willd. were weighed according to appropriate proportions and 22 volumes of 70% ethanol were added for 5 h reflux extraction to concentrate the extract to 0.70, 0.35, and 0.18 g/ml crude drug, which was stored in the dark at 4°C. SASP (Sine [Tianping] Pharmaceutical Co., Ltd., Shanghai, China) and Bupiyichangwan (Guangzhou Chen Li Ji Pharmaceutical Factory Co., Ltd., Guangdong Province, China) were independently ground into powders and filtered through 100-mesh sieves to prepare 0.036 g/ml SASP and 0.16 g/ml Bupiyichangwan suspension in distilled water for later use.

### 2.3. UC Modeling

#### 2.3.1. Protein Extraction and Quantification from Rabbit Colons

Rabbit colon total protein was extracted using radio immunoprecipitation assay (RIPA) lysis buffer (PCode: P0013C, Beyotime Institute of Biotechnology, Shanghai, China) consisting of 50 mM TRIS (pH 7.4), 150 mM NaCl, 1% Nonidet P (NP-40), 0.5% sodium deoxycholate, 0.1% SDS, and protease inhibitors such as sodium orthovanadate, sodium fluoride, and EDTA. First, protease inhibitor phenylmethanesulfonyl fluoride (PMSF) was added to RIPA lysis buffer at a final concentration of 10 mM. Protein was quantified using a bicinchoninic acid (BCA) protein kit (Pro: P0010S, Beyotime Institute of Biotechnology, Shanghai, China) according to the manufacturer's instructions.

#### 2.3.2. Induction of UC Modeling

Equal amounts of Freund's adjuvant (P Code: F5881, Sigma-Aldrich, St. Louis, MO) was mixed with rabbit colon protein to form an antigenic emulsion. Then, 8 mg antigen solution was injected into the toes and groins of rats in the UC model group and different treatment groups and 1.5 ml normal saline was injected into the toes and groins of normal control rats on days 1 and 15. All rats were fasted but had free access to water for 24 h on day 28. Then, animals were anesthetized with isoflurane. Rats in the UC model and different treatment groups were given a 100 mg/kg mixture containing equal volumes of 2,4,6-trinitrobenzenesulfonic acid (TNBS, P Code: 1001910376, Sigma-Aldrich) and 50% ethanol via local enema with a 2 mm diameter catheter for modeling typical UC. Normal controls were given 1 ml 25% ethanol via local enema. After rats received TNBS enema until diarrhea, hematochezia, weight loss, and other symptoms of UC were observed. This confirmed the UC model. Day one for DAI recording was the day of the TNBS enema. A rat disease activity index was recorded every day (Table 1) [17]. The disease index scores of three values were added and divided into three for statistical analysis.

### 2.4. Drug Administration and Sample Collections

Four days after UC modeling, one rat from each group was randomly selected and euthanized to collect colon tissue and evaluate the UC model based on the degree of adhesion of the colon and surrounding tissues and the size of the ulcer. Drug dose was determined by body surface area ratio of rat and human, that is, the dose given to the rat: daily dose per kg of rats = 0.018 × 5 × maximum dose of human. The amount of conversion was used as the moderate-dose Garidisan; twofold of moderate-dose Garidisan was used as the high-dose Garidisan; and 1/2-fold of moderate-dose Garidisan was used as the low-dose Garidisan. After the calculation, rats in the high-dose Garidisan group were given 10 ml/kg of 0.70 g/ml Garidisan solution daily; and rats in the moderate-dose and low-dose Garidisan groups were given 10 ml/kg of 0.35 g/ml and 0.18 g/ml Garidisan solution daily, respectively. The SASP and Bupiyichangwan groups were, respectively, given 10 ml/kg of 0.036 g/ml SASP and 0.16 g/ml Bupiyichangwan suspension daily. Normal controls and UC animals received 10 ml/kg distilled water daily. All rats in each group were treated once a day for 28 days by gavage. Rats in the UC model group had diarrhea and hematochezia throughout the modeling. After the last intragastric administration, all rats were fasted for 24 h, but water was available ad lib. Rats were anesthetized with inhaled isoflurane (2.0%), followed by blood collection (i.e., clotted blood and blood sample stored in anticoagulants) from the heart to collect serum to measure cytokines (from clotted blood) and for flow cytometry (blood samples stored with anticoagulants). Subsequently, taking colon tissues of adjacent healing ulcers from different treatment groups rats were processed three ways: one was postfixed in 2.5% glutaraldehyde for scanning electron microscopy, one was postfixed in 4% paraformaldehyde for morphology detection, and one was snap-frozen and stored at –80°C for molecular biology. Colon tissues dissected from six rats in different treatment groups were raised in cold normal saline, followed by assessment and recording of colon mucosa damage index (CMDI) [18] in a double-blind manner (see calculation standard in Table 2). Scores of two gross morphology values were averaged for statistical analysis.

### 2.5. Morphological Assessment

Colon tissues were fixed in 4% paraformaldehyde, repaired into 0.5 cm, and then underwent conventional paraffin embedding, tissue sectioning into 5 *μ*m thickness, coating the tissue sections on glass slides, heating in a 60°C oven for 2 h, and tissue section storage. During morphological assessment, paraffin tissue sections from each group were stained with conventional H&E and photographed structures under light microscopy (Olympus, BX517-PHD-J11). Two experts double-blind evaluated H&E stained tissue sections according to the evaluation criteria listed in Table 3 to obtain histopathological scores (HS) [19]. Each group had 6 samples, and pictures of five microscopic fields (40x) were taken for each sample. HS scores of different categories were averaged for statistical analysis. Tissue sections from each group were conventionally deparaffinized and hydrated and then stained with Van Gieson's (VG) solution to observe changes in collagen fibers in regenerated tissues before being photographed. Colon tissues fixed in 2.5% glutaraldehyde (0.5 × 0.5 cm) were gradient ethanol dehydrated, critical point dried, and gold sputtered before microvilli were observed on regenerated epithelium surface under SEM (Fei, Quanta, 200 MK2).

### 2.6. Immunofluorescent Assay

Paraffin tissue sections were deparaffinized in xylene solution, rehydrated in gradient ethanol, rinsed in water, and washed three times in 1 × PBS (0.01 mol/l, pH 7.2–7.4) for 5 min each, followed by moderate-to-high-temperature heat retrieving of antigens in the microwave for 10 min. Tissues were cooled to room temperature and rinsed in 1 × PBS three times for 5 min each, and to each was added 50 *μ*l normal goat serum to block nonspecific antigens at 37°C for 30 min in a moisture chamber. After removing excess blocking solution, tissue sections of each sample were independently incubated with 50 *μ*l diluted anti-CD105 and anti-intercellular adhesion molecule (ICAM) 1 primary antibodies (1 : 500, Abcam, Cambridge, MA) at 4°C overnight. After removing the primary antibodies and washing the section in 1 × PBS three times for 5 min each, each tissue section was incubated with 50 *μ*l fluorescent labeled secondary antibody (Beijing Biosynthesis Biotechnology Co., Ltd., Beijing, China) in the dark at 37°C for 30 min. After washing the tissue section in 1 × PBS three times in the dark for 5 min each, each tissue section was wiped dry, followed by adding 50 *μ*l DAPI staining solution, incubation in the dark at 37°C for 10 min, washing in 1 × PBS three times in the dark for 5 min each, wiped drying, and mounting on glass a slide. Slides were observed and photographed under a laser scanning confocal microscope (Fluoview 1000, Olympus). Areas of positively stained cells from each tissue section were analyzed using Image J software. From each group, 3 rats were selected and, for each rat, 2 slides were viewed and, for each slide, 5 horizons were viewed.

### 2.7. ELISA

Blood samples without anticoagulants were stored at room temperature for 2 h and centrifuged at 3,000 rpm for 15 min and serum was collected. Serum interferon- (IFN-) r, interleukin-4 (IL-4), vascular endothelial cell growth factor (VEGF), epidermal growth factor (EGF), and basic fibroblast growth factor (bFGF) were measured according to the manufacturer's instructions on the ELISA kit (Beijing Winter Song Boye Biotechnology Co., Ltd., Beijing, China). For tissue measurements, 200 mg colon tissues stored at −80°C was mixed with ice-cold PBS (100 mg tissues: 1 ml PBS) and homogenized thoroughly with a glass pestle and centrifuged at 3,000 rpm, at 4°C, for 30 min to collect supernatant from tissue homogenate.

### 2.8. Western Blot

Colon tissues stored at −80°C were digested with RIPA lysis buffer (including 10 mM PMSF), followed by centrifugation (14,000 rpm) at 4°C to collect supernatant from the tissue extract for total protein extraction. Extracted total protein from each sample was quantified with the BCA method and separated in conventional SDS-PAGE, which was transferred into a PVDF membrane (ISEQ00010, 0.22 *μ*m, Millipore, USA). After blocking nonspecific antigens on blots, 1 : 800 antivascular endothelial cell growth factor receptor (VEGFR), epidermal growth factor receptor (EGFR), fibroblast growth factor receptor (FGFR) 1, and *β*-actin primary antibodies (Abcam) were incubated with blots at 4°C overnight, followed by washing in 1 × TBST three times at room temperature, 10 min each, and blots were incubated with the corresponding 1 : 3000 diluted secondary antibodies (Beijing Zhong Shan-Golden Bridge Biotechnology Co., Ltd., Beijing, China) on a shaker in the dark at room temperature for 60 min. After 1 × TBST washing at room temperature and exposure to photographic film, the film was imaged using a digital imaging system (Alphalmager 2200 (S-281), Alpha Innotech Co., San Leandro, CA) and analyzed using ImageJ software.

### 2.9. Flow Cytometry

Anticoagulated blood (100 *μ*l) and treated blood (100 *μ*l) from each sample were pipetted to the bottom a flow-cytometry sample tube and appropriate amounts of FITC anti-Rat CD4, PE anti-Rat CD8, APC anti-Rat CD3, PE anti-Rat IL-4, and APC anti-Rat IFN-r antibodies (BioLegend, San Diego, CA) were added and mixed. Then samples were incubated for 20 min at room temperature in the dark. Two microliters of 1 × red cell lysate were add to the samples, mixed well, and centrifuged 5 min at 300 ×g to collect supernatant, which was added to 2 ml cell washing buffer, mixed well, and centrifuged 5 min at 300 ×g to collect supernatant. Subsequently, 500 *μ*l cell washing buffer was add to the supernatant and mixed well before flow cytometry (BD LSRII Flow Cytometry Instrument, BD Corporation).

### 2.10. Statistical Analysis

Data are expressed as means ± SEM. Statistical analysis was performed with Statistical Package for Social Science version 17.0 software (SPSS Inc., Chicago, IL). ANOVA and Mann–Whitney *U* tests were used for analysis. Statistical significance was defined as *P* < 0.05.

## 3. Results

### 3.1. Therapeutic Effect of Garidisan on UC Rats

**Figure 1:**
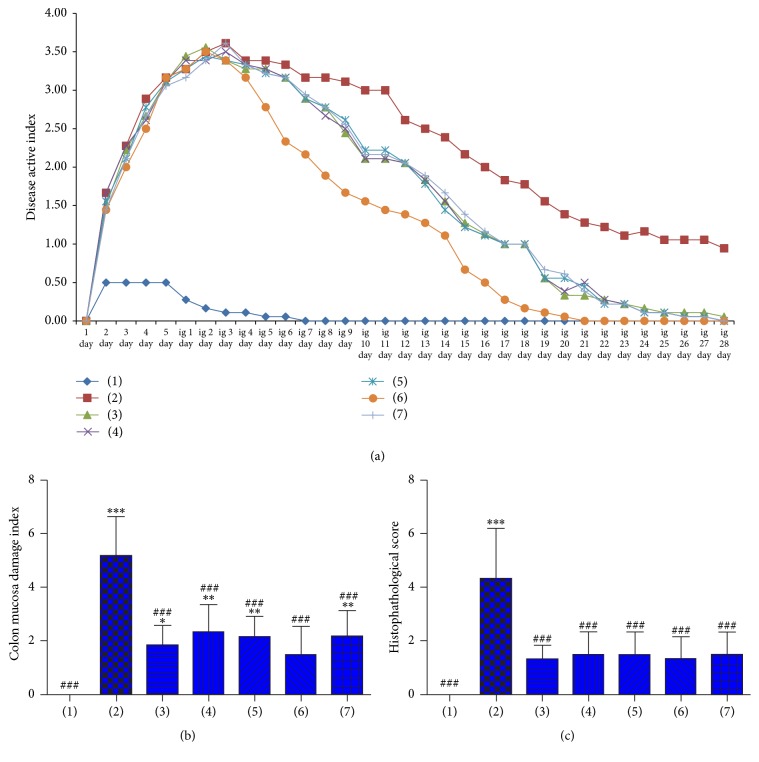
Treatment efficacy of Garidisan in UC rats; (a) DAI for rats after UC modeling; (b) colonic mucosal damage indices for rats in different treatment groups; (c) HS for rats in different treatment groups. Note that (1) represents normal controls; (2) represents the UC model group; (3) represents the low-dose Garidisan group; (4) represents the moderate-dose Garidisan group; (5) represents the high-dose Garidisan group; (6) represents the SASP group; and (7) represents the Bupiyichangwan group; ^*∗*^*P* < 0.05, ^*∗∗*^*P* < 0.01, and ^*∗∗∗*^*P* < 0.001 compared with normal controls; *P* < 0.05, *P* < 0.01, and ^###^*P* < 0.001 compared with the UC model group.

Animal weight, fecal character, and activity in normal controls were normal but animal weight in the UC group declined. Also, UC animals had loose, bloody, or occult blood stools. DAI for UC rats in each groups was elevated and maximized on day 4 after the corresponding treatment. DAI of different treatment and UC model groups began to decline on day 5 after the corresponding treatment. The decline of DAI in the SASP group was the fastest and the first to reach normal. Declines of DAI in other drug treatment groups were similar, and DAI was normal by day 20 after treatment. On day 29 after treatments, DAI of different treatment groups was not significantly different from normal controls but was significantly different from UC rats (Figure 1(a)).

All Garidisan doses reduced CMDI and HS, but declines in CMDI for all Garidisan doses were less than that for the SASP group. Statistical analysis confirmed significant differences in CMDI between low-dose Garidisan and normal controls (*P* < 0.05) and among moderate- and high-dose Garidisan, Bupiyichangwan, and normal controls (*P* < 0.01). No significant difference in CMDI was found between the SASP group and normal controls. Significant differences in CMDI were found between different treatment groups and the UC model group (*P* < 0.001, Figure 1(b)). In addition, no significant difference of HS was found between different treatment groups and normal controls; however, significant differences in HS were found between different treatment groups and the UC model group (*P* < 0.001, Figure 1(c)).

### 3.2. Effects of Garidisan on the Maturity of Mucosal Tissues of UC Rats

**Figure 2:**
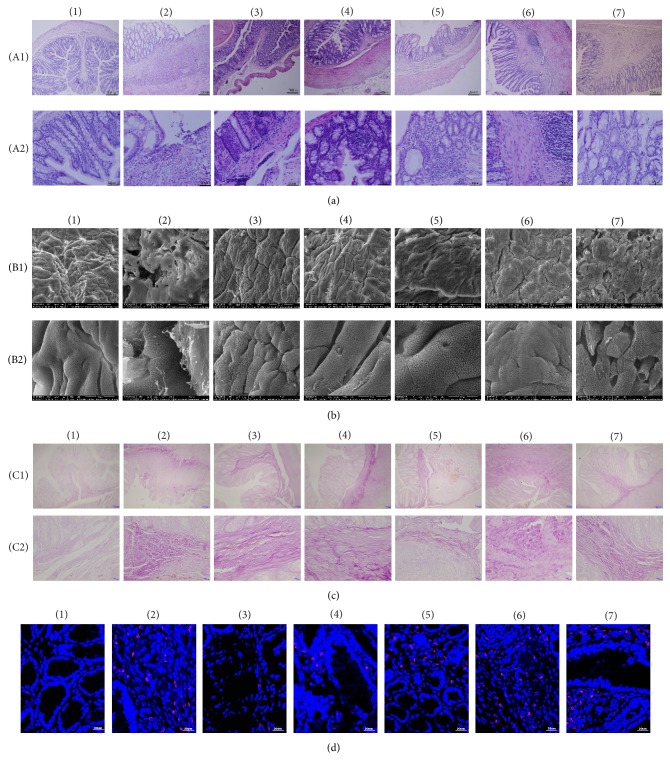
Effects of Garidisan on the degree of maturity of regenerated mucosa. (a) Histological examination of colon tissue of experimental animals (H&E), (A1) representing 100x magnification and (A2) representing 400x magnification; (b) colonic mucosal SEM of experimental animals, (B1) representing 800x magnification and (B2) representing 5,000x magnification; (c) collagen fiber staining (VG staining) in colons of experimental animals, (C1) representing 100x magnification and (C2) representing 400x magnification. Collagen fibers in panel (c) stained purple; (d) ICAM-1 expression in colonic mucosa and submucosal epithelial of experimental animals. Blue DAPI staining represents nuclear staining, and red represents ICAM-1 expression in cy3 staining. Note that (1) represents normal controls; (2) represents the UC model group; (3) represents the low-dose Garidisan group; (4) represents the moderate-dose Garidisan group; (5) represents the high-dose Garidisan group; (6) represents the SAPA group; and (7) represents the Bupiyichangwan group.

The rats of the UC model group had ulcerations in mucosal tissues, whereas mucosal epithelia of UC rats after treatment were intact. The mucosal lamina contained inflammatory cells. Glandular expansion was observed surrounding ulcers in the UC model group. Gland morphology studies of the low- and moderate-dose Garidisan group confirmed many chronic inflammatory cells surrounding the glands. Glands irregularity and some glandular expansions were observed in high-dose Garidisan group. The colorectal gland of the SASP group was similar to the normal group. The colorectal gland of the Bupiyichangwan group also had mild expansion in the mucosa; there were more granulation tissues in submucosa (Figure 2(a)). The Garidisan treatment groups and the SASP group had better recovery of mucosal epithelia than the other treatment groups, with the morphology of the mucosal epithelia being similar as normal controls. The mucosal epithelia of the ulcers in the UC model group were incomplete and had shared boundaries with other tissues. Mucosal surface structures of the Bupiyichangwan group were significantly different from normal controls, with incomplete mucosal surfaces containing many regenerated columnar cells. Cell junctions between nascent epithelia were not fully established, and microvilli on nascent epithelial cells were short and sparse (Figure 2(b)). The deep ulcers in the UC model group contained thick collagen, and collagen fibers of all Garidisan dose groups were significantly smaller and fewer than that of the UC model, SASP, and Bupiyichangwan groups (Figure 2(c)).

According to the results of ICAM-1 immunofluorescence, ICAM-1 was mainly expressed in the connective tissues of the lamina propria and submucosal of regenerated mucosa. ICAM-1 expression was low in normal controls and significantly lower than in the other groups (*P* < 0.001). Drug treatments significantly reduced ICAM-1 expression in regenerated tissues (*P* < 0.001, Figure 2(d), Table 4).

### 3.3. Garidisan Regulates Immune Balance in UC Rats

**Figure 3:**
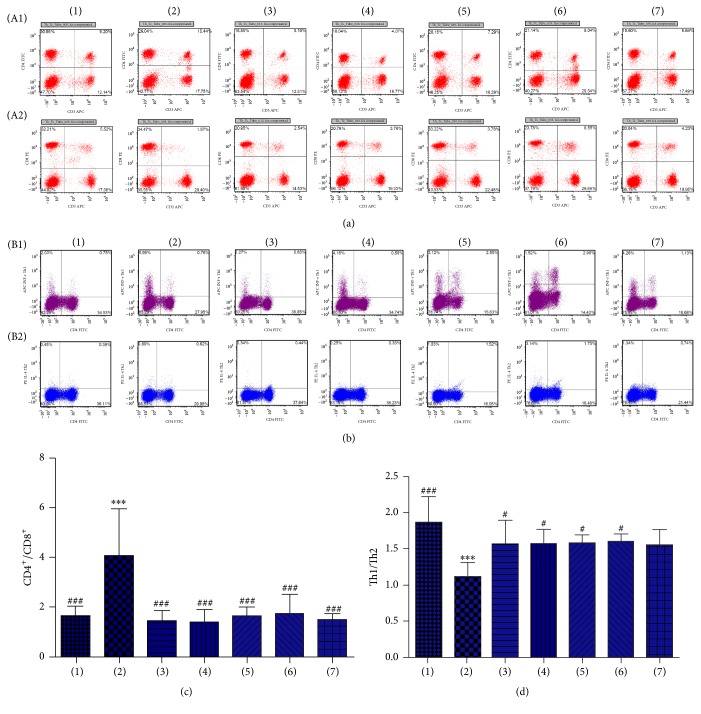
Effects of Garidisan on immune cells in peripheral blood. (a) Flow cytometry for CD3^+^CD4^+^ T and CD3^+^CD8^+^ T cells in peripheral blood of experimental animals; (b) flow cytometry for CD4^+^ Th1 and CD4^+^ Th2 cells in peripheral blood of experimental animals; (c) statistical analysis of CD3^+^CD4^+^ T cells/CD3^+^CD8^+^ T cell ratios; (d) statistical analysis of CD4^+^ Th1/CD4^+^ Th2 ratio. Note that (A1) represents CD3^+^CD4^+^ T cells results of experimental animals; (A2) represents CD3^+^CD8^+^ T cells results of experimental animals; (B1) represents CD4^+^ Th1 cells results of experimental animals; (B2) represents CD4^+^ Th2 cells results of experimental animals; (1) represents normal controls; (2) represents the UC model group; (3) represents the low-dose Garidisan group; (4) represents the moderate-dose Garidisan group; (5) represents the high-dose Garidisan group; (6) represents the SASP group; and (7) represents the Bupiyichangwan group; *P* < 0.05, *P* < 0.01, and ^*∗∗∗*^*P* < 0.001 compared with normal controls; ^#^*P* < 0.05, *P* < 0.01, and ^###^*P* < 0.001 compared with the UC model group.

CD3^+^CD4^+^ T cells, CD3^+^CD8^+^ T cells, and Th1 and Th2 content in peripheral blood of experimental animals as measured with flow cytometry indicated that CD3^+^CD4^+^ T cells/CD3^+^CD8^+^ T cell ratios after treatment were not significantly different from normal controls, whereas CD3^+^CD4^+^ T cells/CD3^+^CD8^+^ T cells ratios after treatment were significantly different from the UC model group (*P* < 0.001, Figures 3(a) and 3(c)). In addition, no significant difference in Th1/Th2 ratios was found between different treatment groups and normal controls, but Th1/Th2 ratios of the UC model group were significantly lower than normal controls (*P* < 0.001). The Th1/Th2 ratio of different treatment groups was significantly elevated compared with the UC model group (Figures 3(b) and 3(d)).

ELISA indicated that IFN-r in peripheral blood of the UC rat group was significantly lower than normal controls and different treatment group (*P* < 0.001). IFN-r in peripheral blood of normal control rats was significantly higher than UC model rats and in different treatment groups (*P* < 0.05). IL-4 in the UC rat model was significantly higher than normal controls and in different treatment groups (*P* < 0.001). IL-4 in peripheral blood of normal controls was significantly lower than for rats in the UC model and in different treatment groups (*P* < 0.01). Results of IFN-r/IL-4 were similar to the results of Th1/Th2 (Table 5).

### 3.4. Effects of Garidisan on the Functional Maturity of Regenerated Mucosa in UC Rats

**Figure 4:**
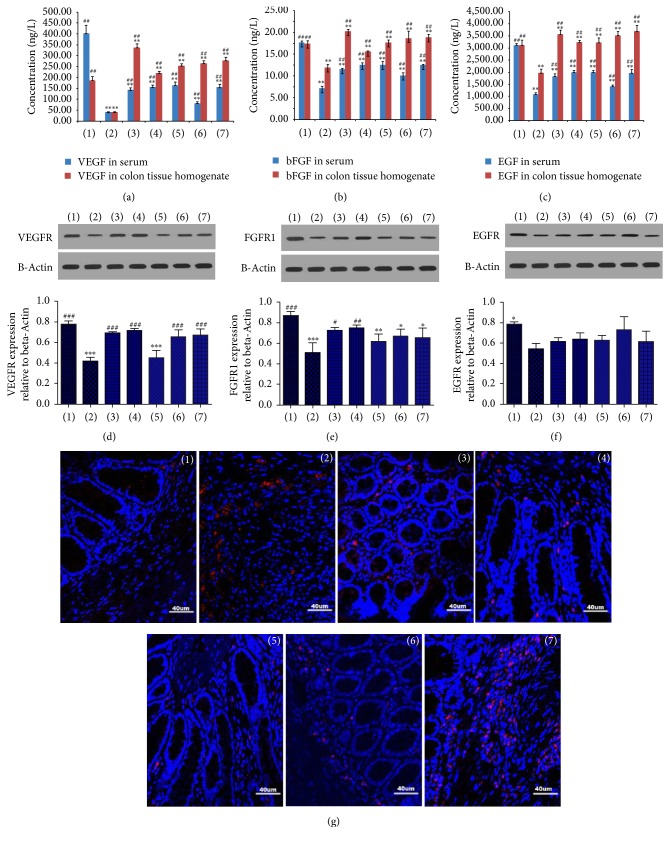
Effects of Garidisan on functional maturity of regenerated mucosa. (a) The concentration of VEGF in serum and colon tissue homogenate; (b) The concentration of bFGF in serum and colon tissue homogenate; (c) The concentration of EGF in serum and colon tissue homogenate; (d) VEGFR expression in colons of experimental animals and statistical analysis; (e) FGFR1 expression in colons of experimental animals and statistical analysis; (f) EGFR expression in colons of experimental animals and statistical analysis; (g) CD105 expression in colons of experimental animals. Blue DAPI staining represents nuclear staining, and red represents CD105 expression in cy3 staining. Note that (1) represents normal controls; (2) represents the UC model group; (3) represents the low-dose Garidisan group; (4) represents the moderate-dose Garidisan group; (5) represents the high-dose Garidisan group; (6) represents the SASP group; and (7) represents the Bupiyichangwan group; ^*∗*^*P* < 0.05, ^*∗∗*  ^*P* < 0.01, and ^*∗∗∗*^*P* < 0.001 compared with normal controls; ^#^*P* < 0.05, ^##^*P* < 0.01, and ^###^*P* < 0.001 compared with the UC model group.

ELISA indicated that VEGF in peripheral blood of experimental rats in different treatment groups was significantly lower than normal controls (*P* < 0.01) and significantly higher than the UC model group (*P* < 0.01). However, VEGF contents in the peripheral blood and the colon tissue homogenate of different treatment groups were significantly higher than the normal control and the UC model groups (*P* < 0.01; Figure 4(a)). EGF in peripheral blood of rats in different treatment groups was significantly lower than the normal control rats (*P* < 0.01) and significantly higher than the UC rats (*P* < 0.01). EGF in colon tissue homogenates of rats in different treatment groups was higher than normal controls, but significantly different. However, EGF in colon tissue homogenates of the rats in different treatment groups was significantly elevated compared with UC model rats (*P* < 0.01; Figure 4(c)). In colon tissue homogenates, bFGF in the low-dose Garidisan group was significantly higher than normal controls (*P* < 0.05) and the UC model group (*P* < 0.01); significant differences in bFGF were not found between remaining treatment groups and normal controls. bFGF in the remaining treatment groups were significantly elevated compared to the UC model group (*P* < 0.01; Figure 4(b)).

Western blot confirmed that there was no significant difference in VEGFR in colon tissue homogenates among the low- and moderate-dose Garidisan, SASP, and Bupiyichangwan groups and normal controls. However, VEGER in colon tissue homogenates of UC rats after different treatments was significantly elevated compared with UC rats (*P* < 0.001, Figure 4(d)). FGFR1 in colon tissue homogenates of all Garidisan dose groups were elevated compared with the UC model group (*P* < 0.05). No significant difference in FGFR1 in colon tissue homogenates was found among the high-dose Garidisan, SASP, and Bupiyichangwan groups and the UC model group (Figure 4(e)). Significant differences in EGFR in colon tissue homogenates were found between normal control and UC model groups. Garidisan, SASP, and Bupiyichangwan increased EGFR expression in colon tissues of UC rats, but these differences had no statistical significance (Figure 4(f)).

Immunofluorescence data showed that CD105 was barely expressed in colon tissues of the normal control rats, whereas CD105 expression in colon tissues of experimental animals was significantly elevated in the other groups (*P* < 0.001). Drug treatments reduced CD105 expression in colon tissue of UC rats, which was lower than the UC model group. Differences in CD105 expression was significant among all Garidisan doses, SASP groups, and the UC model group (*P* < 0.001). There was no significant difference in CD105 expression in colon tissues between the Bupiyichangwan group and the UC model group (Figure 4(g), Table 5).

## 4. Discussion

Recent studies indicate that TNBS-induced chronic ulcer model has three phases: (1) acute inflammation from day 1 to day 14; (2) persistent period of the inflammation (inflammation does not develop during this period) from day 14 to day 35; and (3) chronic inflammation from day 35 to day 49 [20]. We used immunization and TNBS to induce UC in a rat model and the UC model group continuously had ulcerations throughout the experimental processes (from TNBS induction to the end of the experiments in a total of 32 days). With disease development, acute inflammatory cells, neutrophils, were reduced in ulcers and their release of neutrophilic proteolytic enzymes was reduced too. Thus, effusions in granulation tissue could not be completely dissolved and absorbed. The granulation tissue matured into fibrous scar tissue and subsequent fibrosis, thereby reducing the quality of ulcer healing.

Poor quality ulcer healing is a pathological basis of ulcer recurrence [15]. Evaluation of ulcer healing quality included tissue maturity and functional maturity. Tissue maturity included evaluation of indices, such as the integrity of regenerated mucosa epithelia, gland arrangement and condition, and the degree of infiltration of inflammatory cells. Integrity of regenerated mucosal epithelium, proper, nonexpanded gland arrangement and fewer infiltrated inflammatory cells indicate more mature regeneration of mucosal tissues. Functional maturity included expression of growth factors, such as EGF, bFGF, VEGF, and their receptors as well as the circulation of regenerated mucosa [14, 21, 22]. The DAI, CMDI, and HS of UC rats are closely related to UC histopathology results, so they can be used for evaluating therapeutic efficacy [17–19]. DAI, CMDI, and HS data showed that Garidisan, SASP, and Bupiyichangwan could reduce the DAI, CMDI, and HS of UC rats, and these differences were statistically significant. This suggests that Garidisan, SASP, and Bupiyichangwan can be used to treat UC in rats. Results from SEM suggest that Garidisan and SASP repaired the mucosal epithelia and that Bupiyichangwan caused large junctions between mucosal epithelial cells, and surface microvilli of the epithelial cells were short and sparse (Figure 2(b)). This indicates that Garidisan repairs the colon mucosal epithelia in UC rats better than Bupiyichangwan. Data from colonic HS suggested that Garidisan, SASP, and Bupiyichangwan treatments reduced infiltration of inflammatory cells and enhanced tissue maturity of regenerated mucosa. VG staining indicated that Garidisan reduced collagen deposition during ulcer healing and improved ulcer healing quality. Studies indicate an elevation of EGF, VEGF, and bFGF during the acute inflammation phase of ulcer formation. However, growth factors and their activity were reduced when the ulcer changed to a chronic inflammation phase [22, 23]. Our data showed that EGF, VEGF, and bFGF in peripheral blood and colon tissue homogenate of UC model animals were lower than normal controls. Garidisan, SASP, and Bupiyichangwan increased EGF, VEGF, and bFGF in peripheral blood and colon tissue homogenate of UC rats, suggesting an association with the disease progression and differentiation of the mucosal epithelia [24].

Western blot indicated that EGFR, VEGFR, and FGFR1 in the UC model group were significantly lower than in normal controls. VEGFR in low- and moderate-dose Garidisan group, the SASP group, and the Bupiyichangwan group were higher than in the UC model group. In addition, FGFR1 in different treatment groups was higher than in the UC model group. Only low- and moderate-dose Garidisan had statistical significance. EGFR content of the UC model group was lower than in the other groups and was significantly different from normal controls. These differences were similar to differences in growth factors, suggesting that low- and moderate-dose Garidisan could better improve the ulcer healing quality of the UC rats than the high-dose Garidisan. Immunofluorescent data showed that expression of neovascularization marker, CD105, in the UC model group was higher than the other groups, and this may be associated with more granulation tissues in ulcers of the UC model group. CD105 expression in all doses of Garidisan, SASP, and Bupiyichangwan groups was significantly lower than the UC model group but significantly higher than normal controls. These results were similar to the findings of Ippolito's group [25], suggesting that all doses of Garidisan, SASP, and Bupiyichangwan promoted blood vessel maturation of regenerated tissues and enhanced ulcer healing quality.

ICAM-1 is a member of immunoglobulin superfamily that binds to the lymphocyte function-associated antigen-1 on lymphocytes and induces immune response, apoptosis, and inflammatory response [26]. ICAM-1 expression is elevated in inflammatory environments [27] and is closely associated with the initiation of inflammation [28, 29]. During the UC active stage, IL-1*β* activates NF-kB through STAT-3 pathway to induce the polarization of ICAM-1 expression, thereby promoting the migration of neutrophils. Some drugs reduce UC by inhibiting ICAM-1 and other adhesion factors [30, 31]. In this study, ICAM-1 expression in the ulcer marginal zone of the UC model group was higher than normal controls, but ICAM-1 expression in after Garidisan and SASP treatment was significantly lower than the UC model group (Figure 2(d)), suggesting that Garidisan inhibited ICAM-1 expression and reduced migration of inflammatory cells, which reduced UC.

CD3 is a marker of mature T lymphocytes, and CD3^+^ T lymphocytes are classified into CD4^+^ helper T lymphocytes (T helper, Th) and CD8^+^ killer T lymphocytes according to their surface markers. CD3^+^CD4^+^ helper T lymphocytes enhance phagocyte-mediated anti-infective defense and B cell-mediated humoral immune responses. CD3^+^CD8^+^ T cells specifically and directly kill target cells. Flow cytometry showed that the ratio of CD3^+^CD4^+^ T lymphocytes and CD3^+^CD8^+^ T lymphocytes in the UC model group was higher than normal controls, which was consistent with the findings of Postovalova's group [32, 33]. In contrast, different doses of Garidisan, SASP, and Bupiyichangwan reduced the ratio of CD3^+^CD4^+^ T lymphocytes and CD3^+^CD8^+^ T lymphocytes in peripheral blood of UC rats. CD4^+^ T lymphocytes are of a variety of subtypes: for example, Th1, Th2, Th17, and regulatory T lymphocytes (Treg). Previous studies indicate that an imbalance of Th1/Th2 in UC patients and some drugs (e.g., infliximab) can treat UC by regulating this balance [6]. Other studies suggest that increased Th2 but not Th1 occurs in UC patients [34]. We found that the Th1/Th2 ratio was significantly reduced in peripheral blood of UC rats and Garidisan and other drug treatments enhanced this ratio by regulating the balance in peripheral blood of UC rats to varying degrees. In addition, ELISA confirmed an imbalance in Th1-secreted cytokines and Th2-secreted cytokines in peripheral blood of UC rats. These changes were similar to the results of Th1/Th2, suggesting that Garidisan regulated immune cell balance, Th1 and Th2, and their secreted cytokines to achieve a treatment effect.

## 5. Conclusion

All doses of Garidisan enhanced tissue maturation of regenerated mucosa by reducing infiltration of inflammatory cells and collagen formation and improving the integrity of regenerated mucosa and gland maturity in UC ulcers. By promoting maturation of new blood vessels and elevating expression of growth factors and their receptors, Garidisan promoted functional maturity of regenerated tissues. Garidisan likely improved the quality of ulcer healing by reducing ICAM-1 expression and modifying the balance of CD3^+^CD4^+^ T cells/CD3^+^CD8^+^ T cells, Th1/Th2, and their secreted cytokines.

## Figures and Tables

**Table 1:** Scoring of disease activity index.

Weight loss (%)	Stool consistency	Occult/gross bleeding	Score
Normal	Normal	Normal	0
1∼5			1
5∼10	Loose stools	Hemoccult+	2
10∼20			3
>20	Diarrhea	Gross bleeding	4

*Note*. Normal stools = well-formed pellets, loose stools = pasty and semiformed stools which do not stick to the anus, and diarrhea = liquid stools that stick to the anus.

**Table 2:** Colonic mucosal damage index scoring criteria.

Gross morphologies	Symptoms	Score
Correlation with the surrounding tissues during sampling	No adhesion	0
	Mild adhesion (colon and other tissues were easily peeled)	1
	Serious adhesion	2

Inflammation and ulceration	No ulceration and inflammation	0
	Local congestion and no ulceration	1
	1 ulcer without congestion or thickening of intestinal wall	2
	1 ulcer with inflammation	3
	≥2 ulcers with inflammation	4
	>2 ulcers and >1 cm inflamed area	5
	Ulcers with >2 cm inflamed area and increasing 1 score for each additional 1 cm lesion	6–8

**Table 3:** Histological scoring criteria.

	Score 0	Score 1	Score 2
Acute inflammatory cell infiltration	No	Mild increase	Severe increase
Chronic inflammatory cell infiltration	No	Mild increase	Severe increase
Submucosal oedema	No	Patchy	Confluent
Ulcer formation	No	Yes	

**Table 4:** Statistical analysis of ICAM-1 and CD105 expression in regenerated mucosa (M ± SEM, *n* = 8).

	ICAM-1	CD105
Normal control group	181.88 ± 23.62^##^	16.75 ± 11.26^###^
UC model group	3100.33 ± 916.65^*∗∗∗*^	2268.44 ± 353.60^*∗∗∗*^
Low-dose Garidisan group	1225.00 ± 215.24^*∗∗∗*###^	1376.50 ± 296.56^*∗∗∗*###^
Moderate-dose Garidisan group	1362.38 ± 258.00^*∗∗∗*###^	591.38 ± 257.68^*∗∗∗*###^
High-dose Garidisan group	976.25 ± 166.67^*∗∗∗*###^	606.88 ± 202.88^*∗∗∗*###^
Sulfasalazine group	1319.75 ± 360.07^*∗∗∗*###^	863.25 ± 420.16^*∗∗∗*###^
Bupiyichangwan group	2179.00 ± 514.59^*∗∗∗*###^	1617.88 ± 450.23^*∗∗∗*###^

*P* < 0.05, *P* < 0.01, and ^*∗∗∗*^*P* < 0.001 compared with normal controls; *P* < 0.05, ^##^*P* < 0.01, and ^###^*P* < 0.001 compared with the UC model group.

**Table 5:** Effect of Garidisan on IFN-r, IL-4, and IFN-r/IL-4 in peripheral blood (M ± SEM, *n* = 6).

Group	IFN-r (ng/L)	IL-4 (ng/L)	IFN-r/IL-4
Normal control	1414.14 ± 118.10^###^	97.26 ± 3.01^###^	14.53 ± 0.88^###^
UC model	814.95 ± 86.41^*∗∗∗*^	166.32 ± 10.60^*∗∗∗*^	4.91 ± 0.55^*∗∗∗*^
Low-dose Garidisan	1070.78 ± 92.88^*∗∗∗*###^	133.01 ± 7.81^*∗∗∗*###^	8.05 ± 0.44^*∗∗∗*###^
Moderate-dose Garidisan	1075.57 ± 121.05^*∗∗∗*###^	122.17 ± 8.93^*∗∗∗*###^	8.79 ± 0.58^*∗∗∗*###^
High-dose Garidisan	1247.53 ± 91.27^*∗∗*###^	136.97 ± 9.09^*∗∗∗*###^	9.14 ± 0.91^*∗∗∗*###^
Sulfasalazine	1165.63 ± 50.22^*∗∗∗*###^	112.27 ± 7.58^*∗∗*###^	10.44 ± 1.06^*∗∗∗*###^
Bupiyichangwan	1297.87 ± 66.35^*∗*###^	135.42 ± 13.50^*∗∗∗*###^	9.65 ± 0.97^*∗∗∗*###^

^*∗*^ *P* < 0.05, ^*∗∗*^*P* < 0.01, and ^*∗∗∗*^*P* < 0.001 compared with normal controls. *P* < 0.05, *P* < 0.01, and ^###^*P* < 0.001 compared with the UC model group.

## Abbreviations

UC: Ulcerative colitis
SASP: Sulfasalazine
TNBS: 2,4,6-Trinitrobenzenesulfonic acid
HE: Hematoxylin and eosin
DAI: Disease activity index
CMDI: Colonic mucosal damage index
HS: Histological scores
VG: Van Gieson
SEM: Scanning electron microscope
ICAM-1: Intercellular adhesion molecule 1
IFN- r: Interferon-r
IL-4: Interleukin-4
VEGF: Vascular endothelial cell growth factor
EGF: Epidermal growth factor
bFGF: Basic fibroblast growth factor
VEGFR: Vascular endothelial cell growth factor receptor
EGFR: Epidermal growth factor receptor
FGFR: Fibroblast growth factor receptor.
